# A Case of External Iliac Artery Injury during Simultaneous Pancreas–Kidney Transplantation Successfully Managed with Resuscitative Endovascular Balloon Occlusion of the Aorta

**DOI:** 10.70352/scrj.cr.26-0381

**Published:** 2026-07-17

**Authors:** Riku Anai, Keizo Kaku, Kodai Shingaki, Hiroshi Noguchi, Kenta Momii

**Affiliations:** 1 Department of Surgery and Oncology, Graduate School of Medical Sciences, Kyushu University, Fukuoka, Fukuoka, Japan; 2 Emergency & Critical Care Center, Kyushu University Hospital, Fukuoka, Fukuoka, Japan

**Keywords:** resuscitative endovascular balloon occlusion of the aorta (REBOA), simultaneous pancreas–kidney transplantation, retransplantation, vascular injury, hemorrhage control

## Abstract

**INTRODUCTION:**

Simultaneous pancreas–kidney transplantation (SPK) is an established treatment for patients with type 1 diabetes mellitus complicated by end-stage renal disease. In retransplantation cases, removal of the existing graft may be required, and severe adhesions around the graft increase the risk of vascular injury. In particular, bleeding from deep vessels can be difficult to control because vascular exposure and clamping are often challenging.

**CASE PRESENTATION:**

A 45-year-old woman with end-stage renal disease secondary to type 1 diabetes mellitus had previously undergone living-donor kidney transplantation. The graft function deteriorated due to treatment-resistant rejection, and she was scheduled for SPK after receiving a deceased donor organ. During removal of the existing renal graft in the left iliac fossa, the external iliac artery was injured, resulting in uncontrolled arterial bleeding that could not be managed by compression, suturing, or vascular clamping. A resuscitative endovascular balloon occlusion of the aorta (REBOA) catheter was inserted via the left femoral artery and used for temporary blood flow control of bleeding from the injured external iliac artery. This stabilized both the hemodynamics and the operative field, allowing successful suture repair of the arterial injury. Subsequently, kidney and pancreas transplantation were completed. The postoperative course was uneventful.

**CONCLUSIONS:**

In SPK with retransplantation, vascular injury may occur during graft removal. Temporary blood flow control using REBOA may be a useful strategy for achieving hemostasis and securing the operative field in cases of localized arterial injury.

## Abbreviations

CHDF
: continuous hemodiafiltration
CK
: creatine kinase
CRP
: C-reactive protein
HbA1c
: hemoglobin A1c
IABO
: intra-aortic balloon occlusion
REBOA
: resuscitative endovascular balloon occlusion of the aorta
SPK
: simultaneous pancreas–kidney transplantation
SpO_2_
: percutaneous arterial oxygen saturation

## INTRODUCTION

Kidney transplantation is the definitive treatment for end-stage renal disease, while pancreas transplantation is performed for patients with type 1 diabetes mellitus. SPK has been established as one of the standard treatments for patients with type 1 diabetes mellitus complicated by end-stage renal disease.^1)^ In general, vascular anastomoses for pancreatic and renal grafts are performed using the iliac vessels, most commonly the external iliac artery and vein.^2)^ In recent years, the number of retransplantation cases has increased due to graft failure after primary transplantation.^3,4)^ In such cases, the existing graft is sometimes removed and retransplantation is performed at the same site. However, severe adhesions frequently develop around the graft, making surgical procedures technically demanding. In particular, removal of the graft carries a high risk of vascular injury, and bleeding from deep vessels can be difficult to control because vascular exposure and clamping are often challenging.

Herein, we report a case of external iliac artery injury during removal of a previous renal graft in SPK, in which temporary blood flow control using REBOA enabled successful hemostasis and completion of the transplantation.

## CASE PRESENTATION

A 45-year-old woman with end-stage renal disease secondary to type 1 diabetes mellitus had previously undergone ABO-incompatible living-donor kidney transplantation in the left iliac fossa from her mother. Hemodialysis had been initiated in January of year X, and kidney transplantation was performed in June of the same year. Approximately 6 years later, she developed rapid deterioration of graft function due to treatment-resistant rejection. She was registered for SPK in year X + 9. After graft failure, hemodialysis was reinitiated in April of year X + 11. In July of year X + 11, a deceased donor organ became available, and she was admitted for SPK.

Her past medical history included appendectomy, iliac bone grafting for spondylitis, cesarean section, and kidney transplantation. The patient had previously undergone iliac bone grafting for spondylitis; however, this procedure was not considered to be directly related to the left iliac fossa operative field in the present surgery. The dense adhesions encountered during the present operation were considered mainly attributable to the previous kidney transplantation. The initial living-donor kidney transplantation had been performed through an extraperitoneal approach to the left iliac fossa, with the renal artery and vein anastomosed end-to-side to the left external iliac artery and vein, respectively. On admission, her body temperature was 35.5°C, blood pressure was 188/94 mmHg, and pulse rate was 64 beats per minute. Laboratory findings were as follows: white blood cell count, 10080/μL; red blood cell count, 5.26 × 10^6^/μL; hemoglobin, 14.9 g/dL; platelet count, 202000/μL; blood urea nitrogen, 39 mg/dL; creatinine, 8.83 mg/dL; amylase, 71 U/L; lipase, 20 U/L; HbA1c, 6.3%; CRP, 1.0 mg/dL; sodium, 136 mmol/L; potassium, 4.7 mmol/L; chloride, 99 mmol/L; C-peptide, 0.1 ng/mL; anti-glutamic acid decarboxylase antibody, negative; and anti-IA-2 antibody, negative.

CT revealed a transplanted kidney in the left iliac fossa, with the renal artery anastomosed to the left external iliac artery and the renal vein to the left external iliac vein (**Fig. 1**). The deceased donor was a 45-year-old man (height, 160 cm; weight, 77 kg; BMI, 30.1 kg/m^2^) who had died of head trauma, without cardiopulmonary arrest. His HbA1c was 5.2%, and serum creatinine was 0.91 mg/dL.

**Fig. 1:**
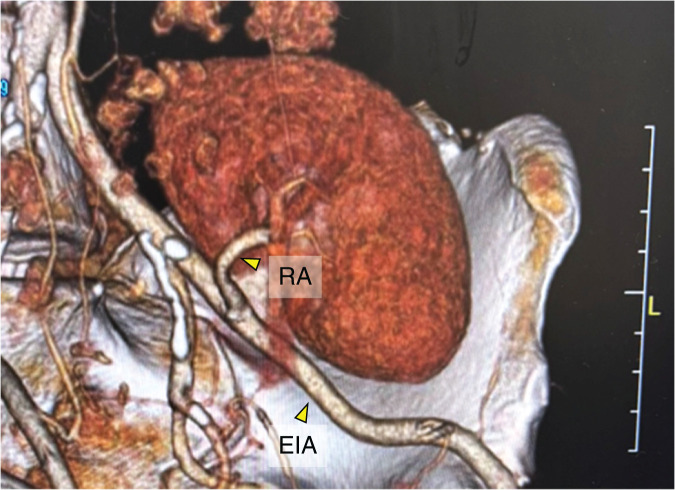
3D CT demonstrating the previously transplanted kidney in the left iliac fossa. The RA is anastomosed end-to-side to the left EIA. EIA, external iliac artery; RA, renal artery

Surgery was initiated with kidney transplantation in the left iliac fossa, followed by pancreas transplantation. Because this was a retransplantation case, removal of the existing renal graft in the left iliac fossa was performed first. In the previous transplantation, the renal vein had been anastomosed to the left external iliac vein and the renal artery to the left external iliac artery in an end-to-side fashion. A 15-cm oblique incision was made in the left lower abdomen. Because severe adhesions were anticipated in the retroperitoneal space, a transperitoneal approach was selected. Dissection and taping of the left external iliac artery were attempted; however, these were abandoned due to dense adhesions around the vessel. Dissection was then continued around the renal graft toward the hilum. During medial dissection of the graft using electrocautery, the external iliac artery was injured, resulting in massive arterial bleeding. Because the bleeding occurred in a deep operative field, vascular clamping was not feasible, and hemostasis by manual compression and suturing was difficult.

While digital compression was applied to control bleeding, an emergency physician was requested to insert an REBOA using a RESCUE BALLOON 7-Fr catheter (Tokai Medical Products, Kasugai, Japan) via the left femoral artery. REBOA placement and management were performed by an emergency physician in cooperation with the transplant surgeons. REBOA placement and balloon inflation were performed under intraoperative fluoroscopic guidance. The guidewire and catheter positions were confirmed fluoroscopically, and the balloon was inflated carefully while monitoring the operative field and the hemodynamic response. The balloon was advanced to the external iliac artery and used to obtain temporary flow control for bleeding from the injured segment (**Fig. 2**). The diameter of the left external iliac artery measured on preoperative CT was 8 mm, and the balloon was inflated with approximately 6 mL of saline (**Fig. 3**). Although proximal balloon occlusion is generally preferable, it was insufficient in this case because distal backflow persisted and the previous renal graft obstructed exposure of the injured external iliac artery. Therefore, complete temporary flow control was required to complete graft nephrectomy and expose the injury site. The balloon was inflated across the injured segment as an unavoidable salvage maneuver. To reduce the risk of overinflation-related vascular injury, the balloon was inflated gradually and adjusted to the minimum volume necessary for bleeding control, based on tactile feedback from the surgeon’s index finger directly compressing the injury site. During uncontrolled arterial bleeding, the lowest recorded blood pressure was 80/30 mmHg, and temporary hemorrhage control with REBOA rapidly stabilized the hemodynamic status. The graft nephrectomy was then completed as quickly as possible, and after adequate exposure of the injured external iliac artery was obtained, the balloon was repositioned proximally to the injury. The arterial injury was an approximately 5-mm round defect. Because the external iliac artery remained densely adherent and could not be safely taped, vascular clamps were not applied. During suturing, the distal external iliac artery was manually compressed to reduce backflow, and the defect was repaired directly with 5-0 Prolene sutures (Ethicon US, Somerville, NJ, USA). After hemostasis was achieved, the REBOA balloon was gradually deflated and completely removed while monitoring hemodynamic changes and for rebleeding. The balloon was initially inflated proximal to the injured segment for 16 min; however, proximal occlusion alone was insufficient because persistent distal backflow prevented adequate hemorrhage control. After balloon deflation, lower limb blood flow was restored during a 28-min interval. Because the failed renal graft obstructed exposure of the injured external iliac artery, the balloon was subsequently moved across the injured segment and inflated for an additional 58 min to obtain complete temporary flow control. The cumulative balloon occlusion time was 74 min. Systemic heparin was not administered. After balloon deflation, the SpO_2_ waveform of the left lower limb was satisfactory, and backflow from the sheath was confirmed after REBOA removal, suggesting preserved distal flow. No discoloration or ischemic change of the lower limb was observed. The peak lactate level was 3.0 mmol/L at 14 h after the second balloon inflation, and the peak CK level was 176 U/L at 10 h after the second inflation. These findings did not suggest clinically significant lower limb ischemia or ischemia–reperfusion injury.

**Fig. 2:**
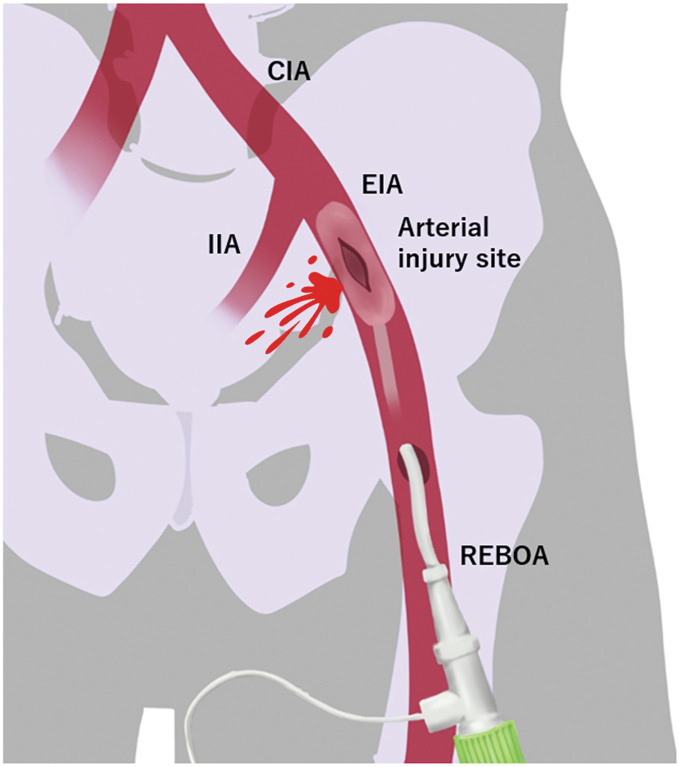
Schematic illustration of temporary endovascular balloon control via the left femoral artery. The REBOA catheter was advanced to the external iliac artery and used to obtain temporary flow control for bleeding from the injured segment. CIA, common iliac artery; EIA, external iliac artery; IIA, internal iliac artery; REBOA, resuscitative endovascular balloon occlusion of the aorta

**Fig. 3:**
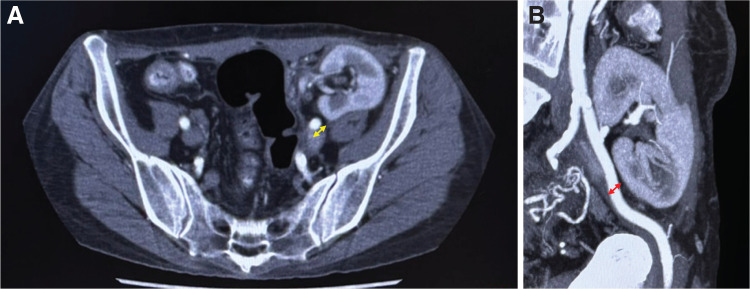
Preoperative CT and balloon sizing reference. (**A**) Axial view and (**B**) coronal view showing the diameter of the external iliac artery (double-headed arrows).

Subsequently, kidney transplantation was performed in the left iliac fossa, followed by pancreas transplantation in the right iliac fossa. The total blood loss was 1840 mL, and the total transfusion requirement was 560 mL of red blood cells and 1440 mL of fresh frozen plasma, with no platelet concentrate transfusion. The operative time was 14 h and 59 min.

Postoperatively, pancreas graft function was favorable, with a rapid decrease in blood glucose levels immediately after reperfusion, resulting in insulin independence. On POD 1, the C-peptide level was 51.95 ng/mL, indicating adequate insulin secretion. Regarding renal graft function, urine output was initially low, and CHDF was initiated on POD 3. The patient was weaned from mechanical ventilation on POD 5, and CHDF was discontinued on POD 6. Hemodialysis was started on POD 8; however, urine output gradually increased, and hemodialysis was discontinued after POD 12. The postoperative course was otherwise uneventful, and the patient was discharged on POD 29.

## DISCUSSION

Kidney transplantation generally provides favorable medium- to long-term graft survival. Recent Japanese registry data have reported a 5-year graft survival rate of 87.8% after deceased-donor kidney transplantation performed between 2010 and 2022, demonstrating favorable medium-term outcomes.^5)^ Nevertheless, chronic graft failure remains an important clinical issue, and retransplantation is sometimes required.^3,4)^ Retransplantation in a previously operated iliac fossa is technically demanding because of dense adhesions, distorted anatomy, and previous vascular anastomoses, and has been associated with an increased risk of vascular complications.^6)^ In the present case, SPK was performed 11 years after the initial kidney transplantation, and removal of the failed graft from the left iliac fossa was required. These factors likely contributed to the difficult dissection and to the external iliac artery injury during graft nephrectomy. In the present case, injury to the external iliac artery during graft removal resulted in uncontrollable bleeding; however, temporary hemostasis using REBOA enabled stabilization of both hemodynamics and the operative field, allowing successful vascular repair and completion of SPK. The unique aspect of the present case is that temporary hemorrhage control was required not only to achieve hemostasis but also to preserve the external iliac artery for subsequent graft implantation. Temporary endovascular balloon control enabled graft nephrectomy, repair and preservation of the external iliac artery, and subsequent completion of SPK. A covered stent graft may be a useful option for iliac artery injury depending on the extent and location of vascular damage. In the present case, the external iliac artery was required as a potential inflow site for renal graft implantation, and stent placement could have reduced the available anastomotic area or complicated subsequent vascular reconstruction. Considering the localized nature of the injury, and the need to complete transplantation, temporary balloon occlusion followed by open suture repair was chosen.

REBOA and IABO were originally introduced for temporary hemorrhage control and circulatory support in patients with severe trauma.^7–9)^ However, their application has recently expanded to selected non-traumatic hemorrhagic conditions and intraoperative bleeding, including refractory upper gastrointestinal bleeding, where they may serve as a bridge to definitive hemostasis by stabilizing hemodynamics and improving the procedural field.^7,10,11)^

A common feature among these reports is that REBOA/IABO is used as a “bridge” to definitive hemostatic procedures. In other words, REBOA is not a definitive treatment itself but rather an adjunctive tool for stabilizing hemodynamics and securing the operative or procedural field. In the present case, REBOA functioned as a bridge to vascular repair of the external iliac artery, enabling control of life-threatening bleeding and continuation of the procedure, consistent with previous reports.

Conceptually, REBOA is designed as an “aortic occlusion balloon,” and its use is generally based on standard zoning, typically Zone 1 or Zone 3, depending on the location of bleeding.^8,9)^ Therefore, advancing the catheter to the level of the external iliac artery, as in the present case, can be regarded as an extrapolated use beyond the conventional zoning concept.

Nevertheless, the principle of achieving hemorrhage control by occluding blood flow proximally to the bleeding site is not inconsistent with the fundamental concept of REBOA. Niimi et al. reported a case of external iliac artery rupture during sheath insertion, in which antegrade blood flow was successfully blocked by placing and inflating a balloon guiding catheter proximal to the rupture site, leading to hemodynamic stabilization followed by successful vascular replacement.^12)^ This report suggests that temporary balloon occlusion proximal to the lesion can be an effective hemostatic strategy even in peripheral arteries such as the external iliac artery.

In the present case, although REBOA was used as the device, the bleeding source was a highly localized injury to the anterior wall of the external iliac artery. By avoiding Zone 1 occlusion, it was possible to minimize ischemia of the abdominal organs. Therefore, this approach can be considered an extension of the concept of “targeted proximal occlusion,” similar to that described by Niimi et al.

On the other hand, balloon occlusion at a more peripheral level carries potential risks, including lower limb ischemia, vascular injury due to smaller vessel diameter, and thrombotic complications. In fact, in the obstetric field, common iliac artery balloon occlusion (CIABO) has been associated with serious complications such as iliac artery thrombosis and lower limb ischemia, highlighting the need for careful consideration of the occlusion site and duration.^13)^

REBOA is generally recommended as a bridge to definitive hemostatic procedures in cases of arterial bleeding below the diaphragm. Accordingly, use outside its established indications should be carefully considered on a case-by-case basis.^8,9)^ Therefore, the use of REBOA beyond standard zoning should be carefully evaluated as an off-label application.

However, in situations such as the present case, where sudden intraoperative injury to the external iliac artery occurs, conventional methods such as direct compression and vascular clamping may be insufficient to control bleeding and secure the operative field. Without rapid vascular repair, continuation of the procedure may be impossible. The use of REBOA in this case was characterized by a clearly identified arterial bleeding source, a lack of alternative methods, a limited objective of temporary circulatory support until vascular repair, and careful control of occlusion time with stepwise deflation and monitoring of ischemic changes. Balloon inflation across an injured arterial segment carries a risk of additional vascular injury and should not be considered a standard technique. However, in the present case, proximal occlusion alone could not provide adequate hemorrhage control because of persistent distal backflow, and the failed renal graft prevented exposure of the injury site. Thus, balloon inflation across the injured segment was performed only as an exceptional salvage maneuver to achieve temporary complete flow control before definitive vascular repair.

Although the cumulative balloon occlusion time was 74 min, this did not represent continuous occlusion. The balloon was first inflated proximally for 16 min, followed by a 28-min interval during which lower limb blood flow was restored, and then inflated across the injured segment for an additional 58 min. This temporary reperfusion interval may have helped reduce the risk of lower limb ischemia. If further prolongation had been required, additional temporary balloon deflation with continued direct digital compression of the injury site was planned to allow transient restoration of lower limb blood flow. Systemic heparin was not administered in the present case because of ongoing uncontrolled arterial bleeding and the urgent need to secure hemostasis. However, when prolonged arterial occlusion is anticipated, systemic heparinization at the time of balloon inflation, followed by protamine reversal after definitive hemostasis, may be preferable to reduce the risk of thrombus formation, while carefully considering the risk of bleeding.

Thus, although the use of REBOA in this case represents an off-label application beyond standard indications, appropriate control of inflation pressure and occlusion time under comprehensive monitoring allowed temporary stabilization of hemodynamics and the operative field, facilitating a smooth transition to definitive hemostatic treatment. Temporary blood flow control using REBOA may therefore be considered a viable therapeutic strategy for localized arterial injury in selected clinical situations. Although prophylactic sheath placement was not performed in the present case, this experience suggests that preoperative sheath placement or preparation for rapid femoral arterial access may be useful in selected high-risk cases, particularly when graft nephrectomy or retransplantation is planned in a previously operated iliac fossa.

## CONCLUSIONS

In retransplantation cases of SPK, the risk of vascular injury is increased due to severe adhesions around the existing graft. In the present case, temporary endovascular balloon control for bleeding from the injured external iliac artery stabilized both hemodynamics and the operative field, enabling successful vascular repair and completion of the procedure. This case suggests that temporary endovascular balloon control using REBOA may be considered a salvage option for localized arterial injury in selected high-risk transplant procedures.

## DECLARATIONS

### Availability of data and materials

Not applicable to this paper as no datasets were generated during this study.
